# Potential pathways and genes expressed in Chrysanthemum in response to early *fusarium oxysporum* infection

**DOI:** 10.1186/s12870-023-04331-7

**Published:** 2023-06-13

**Authors:** Weihao Miao, Yanrong Yang, Mengtong Wu, Gan huang, Lijiao Ge, Ye Liu, Zhiyong Guan, Sumei Chen, Weimin Fang, Fadi Chen, Shuang Zhao

**Affiliations:** 1 College of Horticulture, Nanjing Agricultural University, Nanjing, 210095 China; 2 Key laboratory of landscaping, Ministry of Agriculture and Rural Affairs, Nanjing, 210095 China; 3 Zhongshan Biological Breeding Laboratory, No.50 Zhongling Street, Nanjing, 210014 Jiangsu PR China

**Keywords:** Chrysanthemum, Fusarium wilt, *Fusarium oxysporum*, RNA-seq, WRKY

## Abstract

**Background:**

Chrysanthemum Fusarium wilt is a common fungal disease caused by *Fusarium oxysporum*, which causes continuous cropping obstacles and huge losses to the chrysanthemum industry. The defense mechanism of chrysanthemum against *F. oxysporum* remains unclear, especially during the early stages of the disease. Therefore, in the present study, we analyzed chrysanthemum ‘Jinba’ samples inoculated with *F. oxysporum* at 0, 3, and 72 h using RNA-seq.

**Results:**

The results revealed that 7985 differentially expressed genes (DEGs) were co-expressed at 3 and 72 h after *F. oxysporum* infection. We analyzed the identified DEGs using Kyoto Encyclopedia of Genes and Genomes and Gene Ontology. The DEGs were primarily enriched in “Plant pathogen interaction”, “MAPK signaling pathway”, “Starch and sucrose metabolism”, and “Biosynthesis of secondary metabolites”. Genes related to the synthesis of secondary metabolites were upregulated in chrysanthemum early during the inoculation period. Furthermore, peroxidase, polyphenol oxidase, and phenylalanine ammonia-lyase enzymes were consistently produced to accumulate large amounts of phenolic compounds to resist *F. oxysporum* infection. Additionally, genes related to the proline metabolic pathway were upregulated, and proline levels accumulated within 72 h, regulating osmotic balance in chrysanthemum. Notably, the soluble sugar content in chrysanthemum decreased early during the inoculation period; we speculate that this is a self-protective mechanism of chrysanthemums for inhibiting fungal reproduction by reducing the sugar content in vivo. In the meantime, we screened for transcription factors that respond to *F. oxysporum* at an early stage and analyzed the relationship between *WRKY* and DEGs in the “Plant-pathogen interaction” pathway. We screened a key *WRKY* as a research target for subsequent experiments.

**Conclusion:**

This study revealed the relevant physiological responses and gene expression changes in chrysanthemum in response to *F. oxysporum* infection, and provided a relevant candidate gene pool for subsequent studies on chrysanthemum Fusarium wilt.

**Supplementary Information:**

The online version contains supplementary material available at 10.1186/s12870-023-04331-7.

## Background

Chrysanthemum, a perennial plant belonging to the family Asteracae, has been cultivated for thousands of years in China and has a high cultural value [1]. Chrysanthemum follows rose in the cut flower trade and holds a prominent position in the global flower market [2]. Chrysanthemum Fusarium wilt is a common soil-borne disease of chrysanthemum that occurs throughout its growth period. *Fusarium oxysporum* is the primary pathogen causing Chrysanthemum Fusarium wilt. It is a facultative parasitic fungus that can survive in plants and soils. *F. oxysporum* invades the roots of plants, resulting in blockage of vascular bundles, and causes water shortage in the above-ground parts of plants, resulting in wilting, yellowing, and eventually death of plant leaves [3–5]. Additionally, *F. oxysporum* can secrete a pathogenic toxin during the process of infection known as fusaric acid, which has allelopathic effects on plants and affects their healthy growth [6]. *F. oxysporum* spreads widely and causes more than 100 plant diseases in a wide host range, such as cucumber, cotton, banana, and flowers [7–9]. In recent years, with the continuous expansion of chrysanthemum planting area, *F. oxysporum* has spread rapidly in the soil and among chrysanthemums, causing frequent obstacles to the continuous cropping of chrysanthemum and a severe reduction in its production.

Plants have evolved to rely on their defense systems to resist biotic stress. After the recognition of pathogen associated molecular patterns (PAMP) by pattern recognition receptors on plant cell membranes, PAMP-triggered immunity is activated. When pathogenic bacteria secrete effectors into the plant, the plant can rely on its resistance proteins to activate effector-triggered immunity and effectively inhibit the spread of pathogens [10–12]. Furthermore, during biological stress, the osmotic adjustment substances accumulated in plants can regulate, protect and scavenge active oxygen and perform other functions. Phenylalanine ammonia-lyase (PAL) and peroxidase (POD) are critical enzymes involved in the biosynthesis of secondary metabolites in plants, and improving the activity of these defense enzymes can increase the stress resistance of plants [13]. More importantly, the defense mechanisms of plants in response to *F. oxysporum* infection have gradually been discovered. When *F. oxysporum* infects tomatoes, vanillin, vanillic acid, and other phenolic metabolites in tomato roots can enhance the deposition of root cell walls, thus delaying the invasion of pathogens [14]. A study on responses of soybean to *F. oxysporum* infection revealed that the changes in secondary metabolites in wild soybean were more significant than those in cultivated soybean, and the disease symptoms were weaker [15]. In addition to plant defense enzymes and secondary metabolites, plant transcription factors (TFs) play an important role in the resistance pathway. Overexpression of *TaNACL-D1* enhances the resistance of wheat plants to Fusarium wilt [16]. In recent studies on cotton Fusarium wilt, IIc WRKY TFs were shown to upregulate the expression of *GhMYC2*-mediated flavonoid synthesis genes, accumulate flavonoid content, and enhance resistance to *F. oxysporum* [17]. However, only few studies have investigated the mechanism of chrysanthemum response to *F. oxysporum* infection. For example, *CmWRKY6-1* and *CmWRKY8-1* in chrysanthemum could respond to *F. oxysporum* infection via the SA pathway [18, 19]. At present, more about the mechanism of chrysanthemum response to *F. oxysporum* infection is still unclear.

The defense mechanisms of plants are complex, and RNA-seq technology can be used to effectively analyze changes in gene expression during the interaction between plants and pathogens. RNA-seq is used to sequence specific species in a specific period to analyze the expression of some genes during this period, providing an important reference for screening key genes [20]. Comprehensive metabolomic and transcriptomic analysis showed that genes related to the flavonoid biosynthesis pathway, IAA, SA, and JA signal transduction pathways were significantly enriched in wheat leaves inoculated with *Fusarium graminearum* [21]. RNA-seq technology to study the effect of *CmWRKY53* on chrysanthemum implied that *CmWRKY53-*mediated aphid sensitivity might be related to secondary metabolites [22]. In the present study, we used RNA-seq technology to analyze the differential expressed genes (DEGs) in chrysanthemum during the early stage of inoculation with *F. oxysporum*, investigate the physiological response and gene expression changes in chrysanthemum in response to *F. oxysporum* infection. We analyzed the changes of related pathways in chrysanthemum after inoculation with *F. oxysporum*, and provided candidate genes for developing new cultivars resistant to Fusarium wilt in chrysanthemum.

## Results

### Morphological identification of *F. oxysporum* and Root Rot of Chrysanthemum

**Fig. 1:**
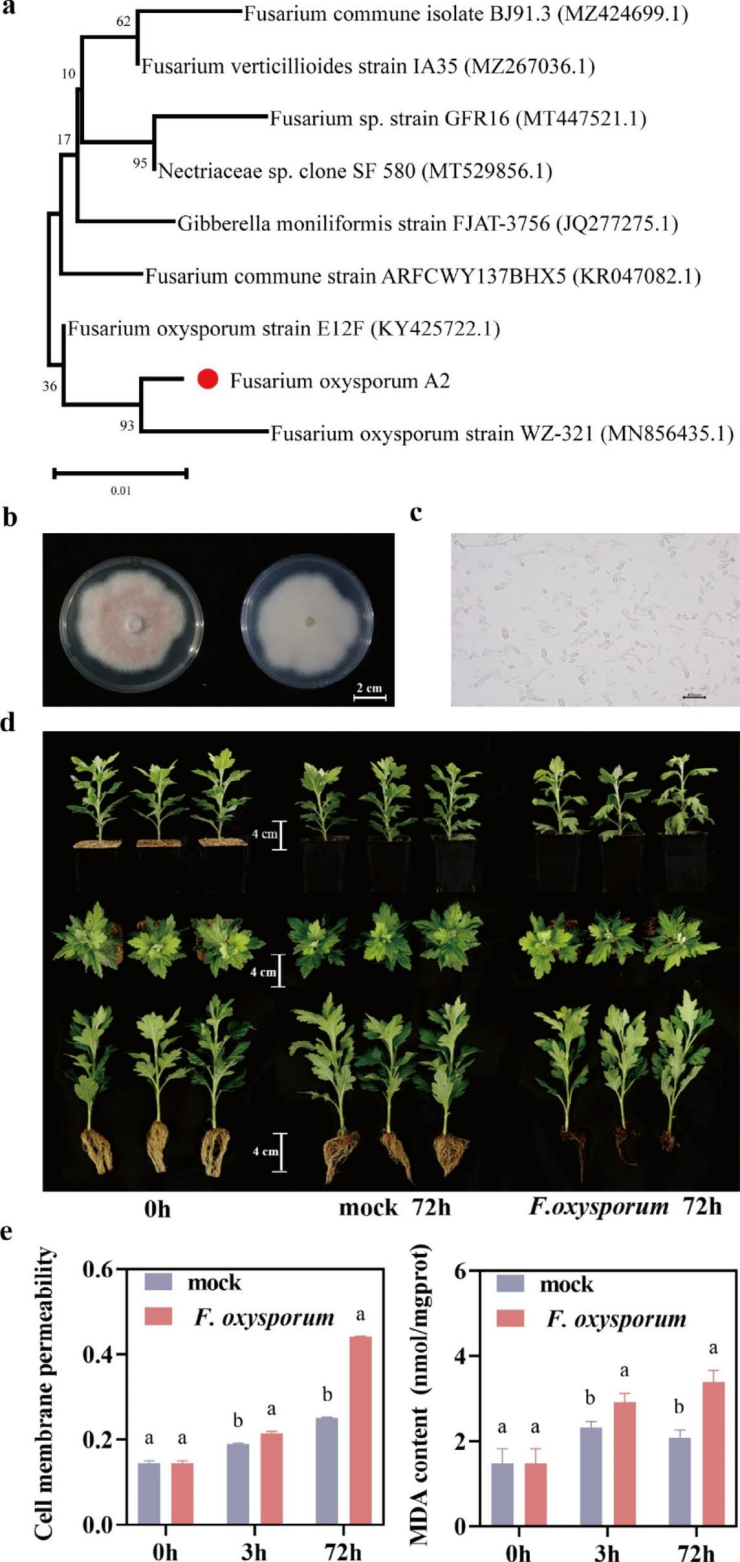
Identification and pathogenic phenotype of *F. oxysporum* A2. **a** Phylogenetic tree of *F. oxysporum* A2, the scale bar is 0.01. **b** Macroscopic observation of the phenotype of *F. oxysporum* A2, the scale bar is 2 cm. **c** Microscopic observation of the phenotype of *F. oxysporum* A2, the scale bar is 100 μm. **d** Phenotypic observation of ‘Jinba’ before and after infection by *F. oxysporum* A2, the scale bar is 4 cm. **e** Changes of cell membrane permeability (Left) and MDA (Right) in ‘Jinba’ after infection by *F. oxysporum* A2

The genomic DNA of the pathogenic fungus was extracted, amplified using PCR, and sequenced. The sequencing results were compared with National Center for Biotechnology Information (NCBI) nucleic acid sequences to establish a phylogenetic tree, which showed that it had the highest homology with *Fusarium oxysporum* strain WZ 321 (Fig. 1a). Furthermore, macroscopic observations revealed a pink fluff filamentous shape on the PDA plate (Fig. 1b). In addition, microscopic observations showed that the spores were curved at both ends (Fig. 1c). Therefore, we named this strain *Fusarium oxysporum* A2.

The ‘Jinba’ cultivar was inoculated with *F. oxysporum*. At 72 h after inoculation, the roots were browned and rotted, and the leaves above the roots of ‘Jinba’ began to wilt slightly (Fig. 1d). The results showed that *F. oxysporum* began to invade the shoot from the root, blocking the vascular bundle and causing a water shortage. Furthermore, we determined the cell membrane permeability and MDA content of ‘Jinba’ before and after inoculation. The results showed that the cell membrane permeability and MDA of ‘Jinba’ were significantly higher than those of the control at 3 and 72 h after inoculation and gradually increased (Fig. 1e).

### Library Construction and sequencing

**Fig. 2:**
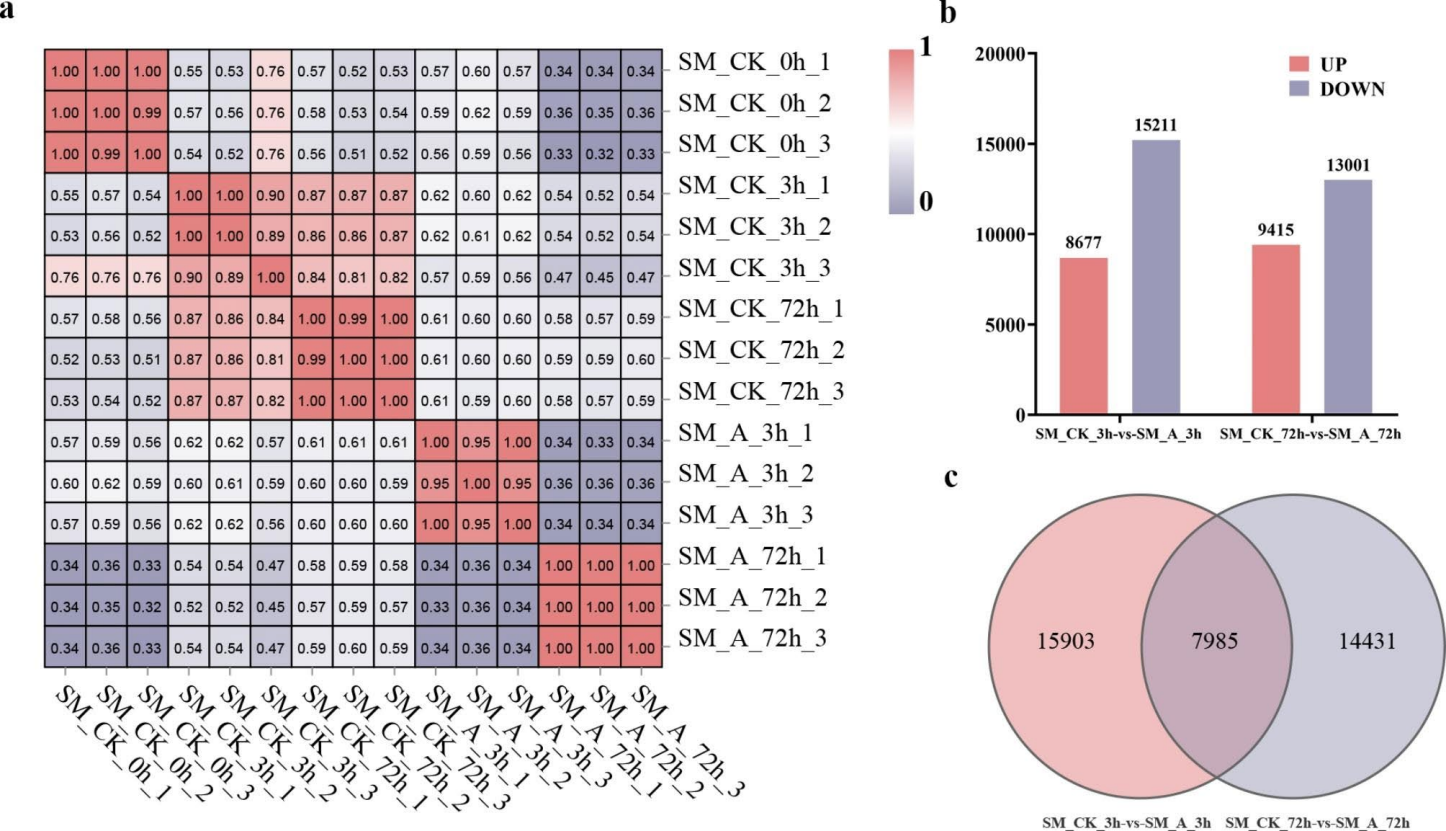
RNA-seq analysis. **a** Correlation among samples. **b** Number of up-and down-regulated DEGs. **c** Venn diagram of 3 h vs. 72 h co-expressed genes

The roots of ‘Jinba’ inoculated with *F. oxysporum* at 0, 3, and 72 h were analyzed using RNA-seq (the control group was named SM-CK-0 h, SM-CK-3 h, and SM-CK-72 h; the experimental group was named SM-A-3 h and SM-A-72 h). 720,233,772 clean data points were obtained, and the percentage of Q30 bases in all samples was ≥ 90% (Additional file 1: Table S1). Correlation analysis showed that the samples within the group were highly correlated (Fig. 2a). All the sequencing results showed that the quality of the sequencing data was reliable and could be used for further analysis. The RNA-seq data has been uploaded to the NCBI SRA database. The current sequencing data were deposited in the NCBI SRA under the accession number PRJNA926886.

### Differentially expressed genes analysis

To identify the genes of ‘Jinba’ that respond to *F. oxysporum* infection in the early stage, we compared the DEGs of the experimental group at 3 h after inoculation and the control group at 3 h without inoculation, as well as the DEGs of the experimental group at 72 h after inoculation with the control group at 72 h without inoculation. Using edgeR, we set the thresholds for log2 fold-change ≥ 1 and FDR (padj) < 0.05. As a result, a total of 8677 genes were downregulated, 15,211 genes were upregulated between 3 h after inoculation and 3 h without inoculation, 9415 genes were downregulated, and 13,001 genes were upregulated between 72 h after inoculation and 72 h without inoculation (Fig. 2b). Among these DEGs, 7985 were co-expressed at both time points (Fig. 2c). Among the 7985 co-expressed genes, we analyzed their expression trends at 0, 3, and 72 h after inoculation. The results showed that 1959 genes were downregulated first and then upregulated, 960 genes were upregulated first and then downregulated, 205 genes were upregulated continuously, and 554 genes were downregulated continuously (Additional file 3: Fig. S1).

As our study aimed to identify the genes and pathways involved in the early response to *F. oxysporum* infection, 7985 co-expressed genes were the focus of our next analysis.

### GO and KEGG Analysis of co-expressed DEGs

**Fig. 3:**
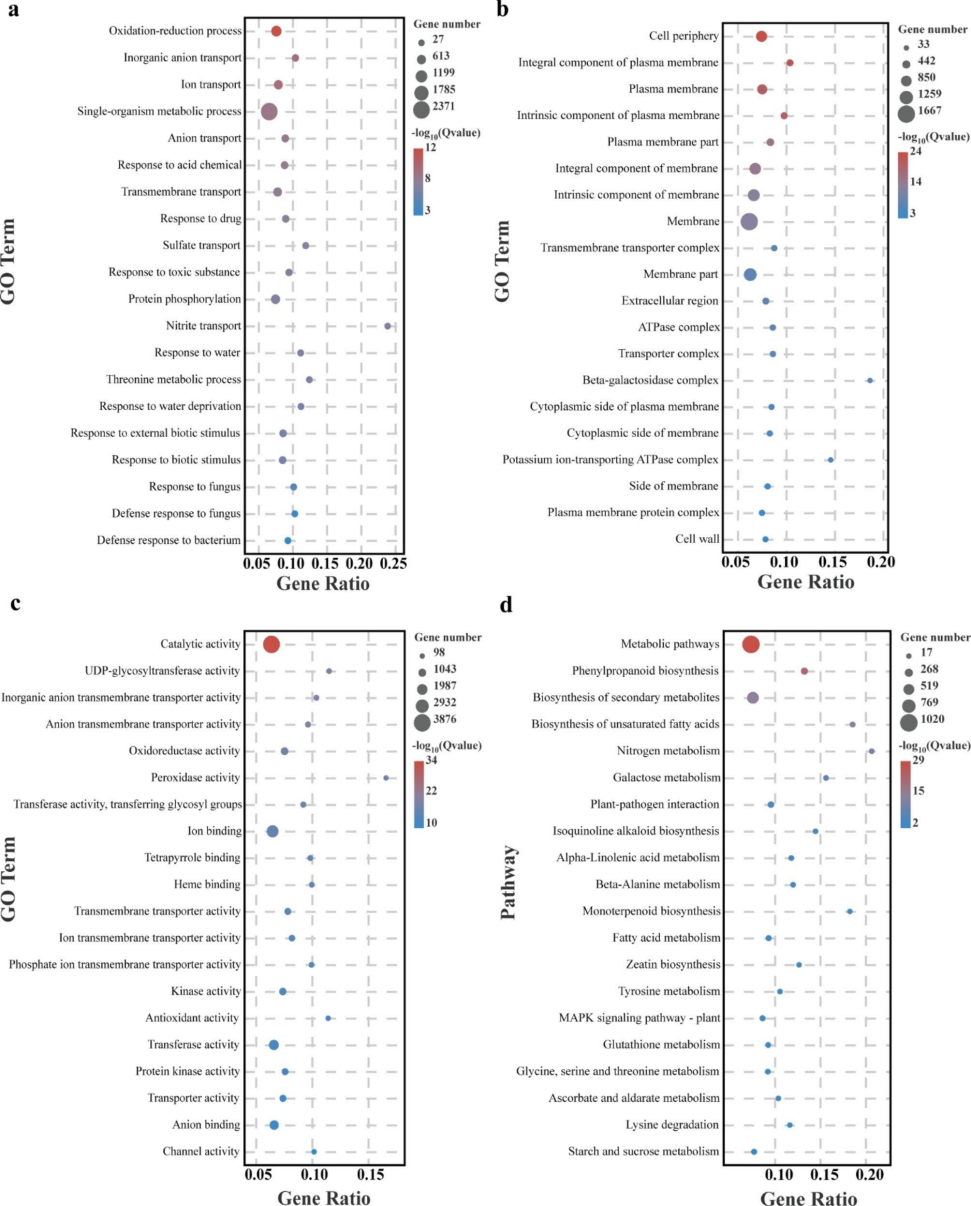
GO and KEGG analysis of co-expressed DEGs. **a** The top 20 GO terms with the most significant Q value in “Biological process”. **b** The top 20 GO terms with the most significant Q value in “Cellular component”. **c** The top 20 GO terms with the most significant Q value in “Molecular Function”. **d** The top 20 KEGG pathways

To explore the relevant functions of the 7985 co-expressed genes, we performed GO analysis. Enriched to “Biological process”, “Cellular component”, and “Molecular Function” with 5295, 847, and 1981 entries, respectively. We analyzed the top20 GO terms that were significantly enriched. In the “Biological process”, several GO terms relating to redox and plant responses to fungi, water, and organisms are significantly enriched: “Oxidation-reduction process”, “Response to water”, “Response to water deprivation”, “Response to external biotic stimulus”, “Response to biotic stimulus”, “Response to oxidative stress”, “Response to fungus”, “Defense response to fungus”, and “Defense response to bacterium” (Fig. 3a). In the “Cellular component”, we found a significant enrichment of GO terms for membrane: “Membrane part”, “Plasma membrane”, “Cytoplasmic side of membrane”, and “Integral component of membrane” (Fig. 3b). In “Molecular Function”, most of the GO terms were related to activity: “Catalytic activity”, “Oxidoreductase activity”, and “Peroxidase activity” (Fig. 3c).

At the same time, we performed KEGG analysis on these DEGs, and 129 KEGG pathways were enriched. In addition, we analyzed the top 20 KEGG pathways, including “Plant-pathogen interaction”, “MAPK signaling pathway”, “Starch and sucrose metabolism”, and “Biosynthesis of secondary metabolites”, which are associated with plant disease resistance (Fig. 3d).

### Secondary metabolites

**Fig. 4:**
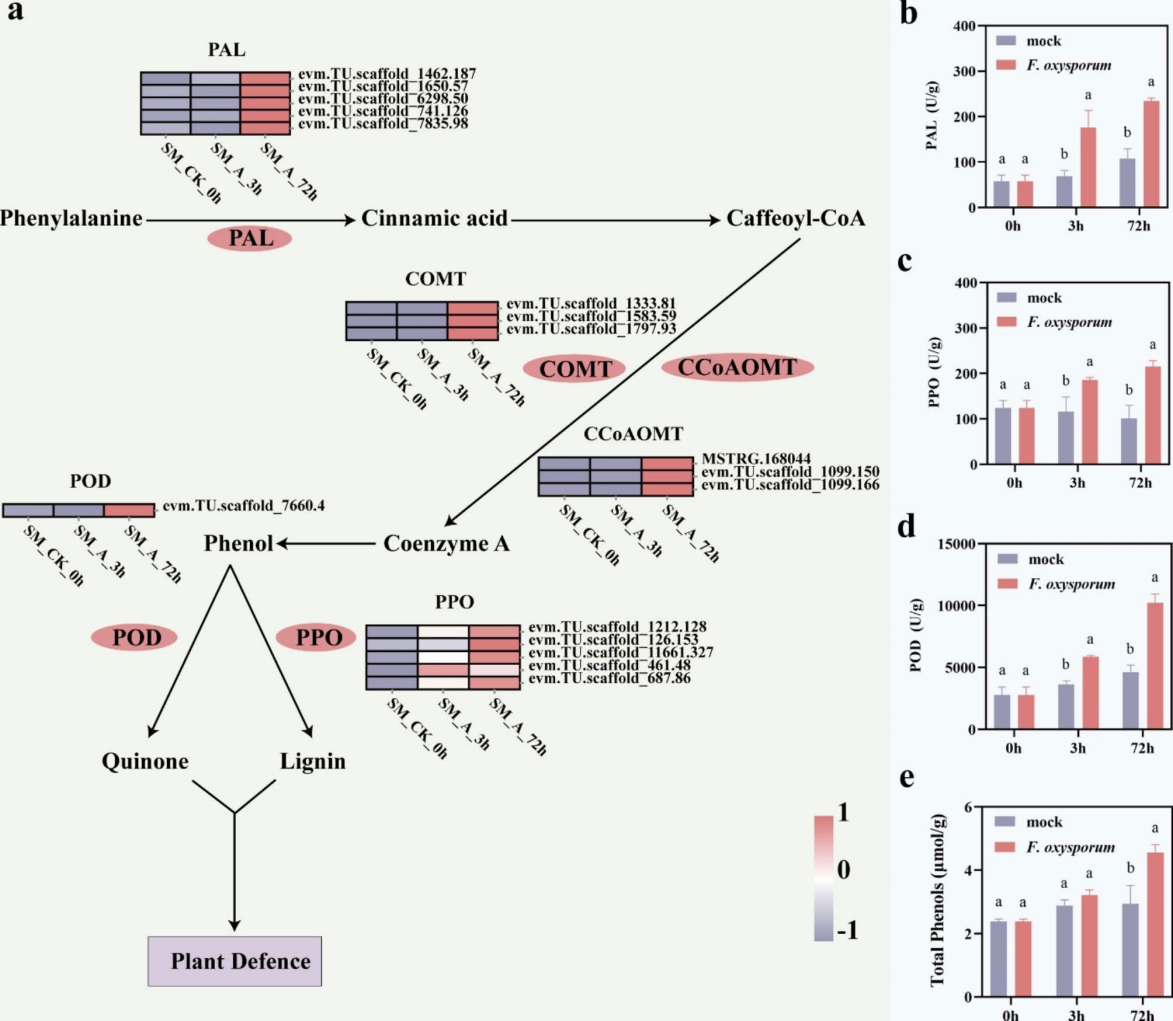
DEGs involved in the “Biosynthesis of secondary metabolites”. **a** Expression analysis of DEGs related to signal transduction pathways of secondary metabolites. Legend normalized for gene expression using z-score; the same scheme applies below. **b** Changes of PAL in ‘Jinba’ after infected by *F. oxysporum*. **c** Changes of PPO in ‘Jinba’ after infected by *F. oxysporum*. **d** Changes of POD in ‘Jinba’ after infected by *F. oxysporum*. **e** Changes of total phenols in ‘Jinba’ after infected by *F. oxysporum*

As we enriched the 3 and 72 h co-expressed DEGs to the “Biosynthesis of secondary metabolites” pathway through the KEGG analysis, we analyzed the mechanisms concerning the plant response to pathogen infection from the DEGs of the “Biosynthesis of secondary metabolites” pathway (Fig. 4a). The secondary metabolites of plants contain phenolics, terpenoids, and nitrogenous compounds that play important roles in insecticide, antibacterial, and antioxidant resistance [23]. Within 72 h of *F. oxysporum* infection, five *PAL* (evm.TU.scaffold_1462.187, evm.TU.scaffold_1650.57, evm.TU.scaffold_6298.50, evm.TU.scaffold_741.126, evm.TU.scaffold_7835.98), three *COMT* (evm.TU.scaffold_1333.81, evm.TU.scaffold_1583.59, evm.TU.scaffold_1797.93), *CCoAOMT* (MSTRG.168,044, evm.TU.scaffold_1099.150, evm.TU.scaffold_1099.166), *PPO* (evm.TU.scaffold_1212.128, evm.TU.scaffold_126.153, evm.TU.scaffold_11661.327, evm.TU.scaffold_461.48, evm.TU.scaffold_687.86), and one *POD* (evm.TU.scaffold_7660.4) were upregulated. At the same time, to analyze the changes in secondary metabolites more intuitively after inoculation, we determined the enzyme activity of PAL, polyphenol oxidase (PPO), and POD, which are related to phenol synthesis in plants, and the total phenol content. The results showed that the enzyme activities of PAL, PPO, and POD in ‘Jinba’ increased after inoculation with *F. oxysporum*, and the total phenol content also increased accordingly (Fig. 4b, c, d, e). In conclusion, within 72 h after inoculation of ‘Jinba’, the infection of *F. oxysporum* affected the genes related to the “Biosynthesis of secondary metabolites” pathway, thus changing the content of enzymes related to phenolic synthesis and total phenolic content in ‘Jinba’.

### Plant osmotic substances

**Fig. 5:**
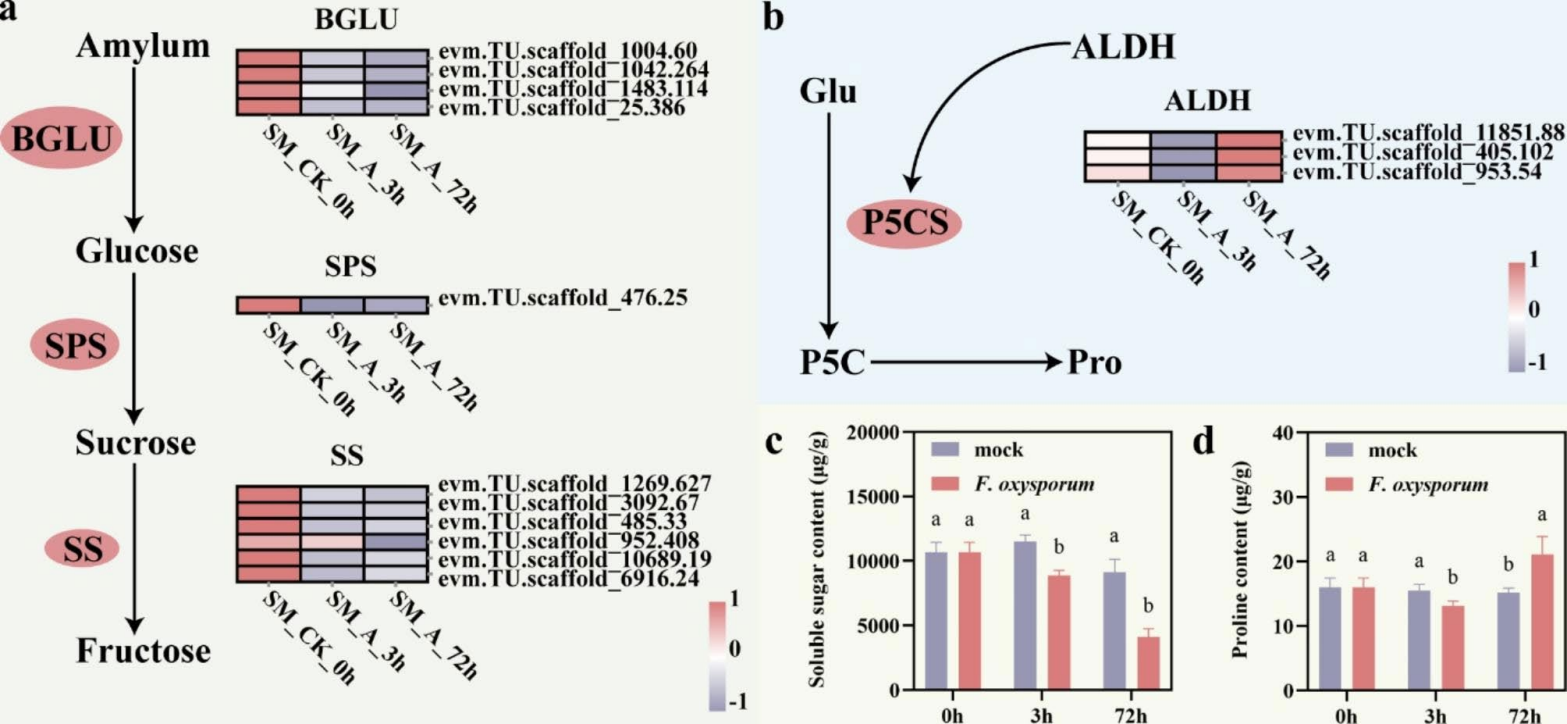
DEGs involved in plant osmotic substances. **a** Expression analysis of DEGs related to signal transduction pathways of starch and sucrose metabolism. **b** Expression analysis of DEGs related to signal transduction pathways of proline metabolism. **c** Changes of soluble sugar in ‘Jinba’ after infected by *F. oxysporum*. **d** Changes of proline in ‘Jinba’ after infected by *F. oxysporum*

When plants are subjected to water or osmotic stress, they protect themselves by regulating osmotic substances, such as soluble sugars and proline [24]. Changes in these osmotic substances are essential mechanism for the survival of plants in adverse environments. To explore the changes in osmotic substances in response to *F. oxysporum* infection, we analyzed the DEGs of the co-expressed gene-related pathways at 3 and 72 h after inoculation (Fig. 5a, b). In the “Starch and sucrose metabolism” pathway, four *BGLU* (evm.TU.scaffold_1004.60, evm.TU.scaffold_1042.264, evm.TU.scaffold_1483.114, evm.TU.scaffold_ 25.386), one *SPS* (evm.TU.scaffold_476.25), and six *SS* (evm.TU.scaffold_1269.627, evm.TU.scaffold_3092.67, evm.TU.scaffold_485.33, evm.TU.scaffold_952.408, evm.TU.scaffold_10689.19, evm.TU.scaffold_6916.24) were significantly decreased within 72 h of inoculation compared to pre-inoculation. In the “proline and arginine metabolism” pathway, three *ALDH* (evm.TU.scaffold_11851.88, evm.TU.scaffold_405.102, evm.TU.scaffold_953.54) were significantly decreased at 3 h after inoculation and significantly increased at 72 h after inoculation.

Further, we measured the changes in the content of these osmotic substances in ‘Jinba’ before and after inoculation. We found that soluble sugar content decreased significantly after inoculation (Fig. 5c). Proline levels transiently decrease at 3 h, followed by a significant increase at 72 h (Fig. 5d). The results showed that the genes related to the “Starch and sucrose metabolism” and “proline and arginine metabolism” pathways were affected by the invasion of *F. oxysporum* in ‘Jinba’ within 72 h after inoculation, thereby changing the content of soluble sugar and proline in ‘Jinba’.

### TF analysis

**Fig. 6:**
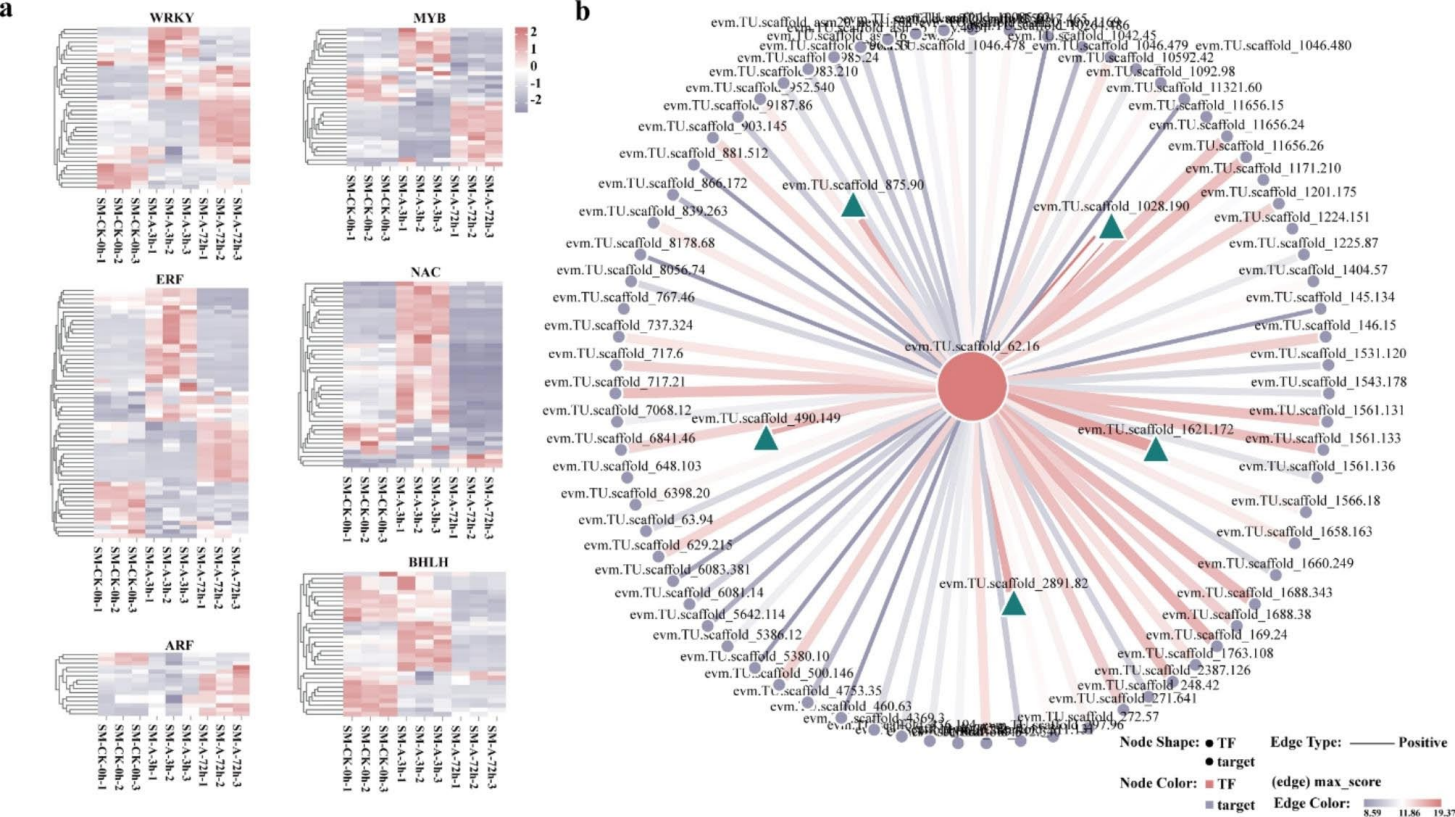
Transcription Factor Analysis. **a** Transcription factor heat map analysis. **b** Targeting analysis between evm.TU.scaffold_62.16 and Pathogen-Related Genes. The red circle indicates evm.TU.scaffold_62.16, purple circles indicate target genes, and green triangles represent high-scoring target genes

TFs have attracted much attention in the plant response to pathogen infection. In this study, 38 *WRKY*, 32 *MYB*, 42 *NAC*, 58 *ERF*, 15 *ARF*, and 32 *bHLH* genes were identified in the 3 and 72 h co-expressed DEGs (Fig. 6a). WRKY family members often play an important role in plant disease resistance pathways [25]. To further investigate the mechanism of WRKY response to *F. oxysporum* in ‘Jinba’, we analyzed the WRKY-target genes containing the “Plant-pathogen interaction” pathway (Additional file 2: Table S2). Among these WRKYs, evm.model.scaffold_62.16 had the highest homology score with CmWRKY6. Song et al. (2014) found that *CmWRKY6* is involved in the response to *F. oxysporum* infection in chrysanthemums [26]. Their conserved structural domains were highly similar comparing the protein sequences of evm.TU.scaffold_62.16 and CmWRKY6 (Additional file 3: Fig. S2). At the same time, we mapped the association network between evm.TU.scaffold_62.16 and target genes containing the “Plant-pathogen interaction” pathway (Fig. 6b). We found that evm.TU.scaffold_62.16 with *CNGC* (evm.TU.scaffold_1028.190) and *CPK* (evm.TU.scaffold_1621.172, evm.TU.scaffold_875.90, evm.TU.scaffold_490.149 and, evm.TU.scaffold_2891.82) have a high potential target relationship.

### Confirmation of DEGs using qRT-PCR

**Fig. 7:**
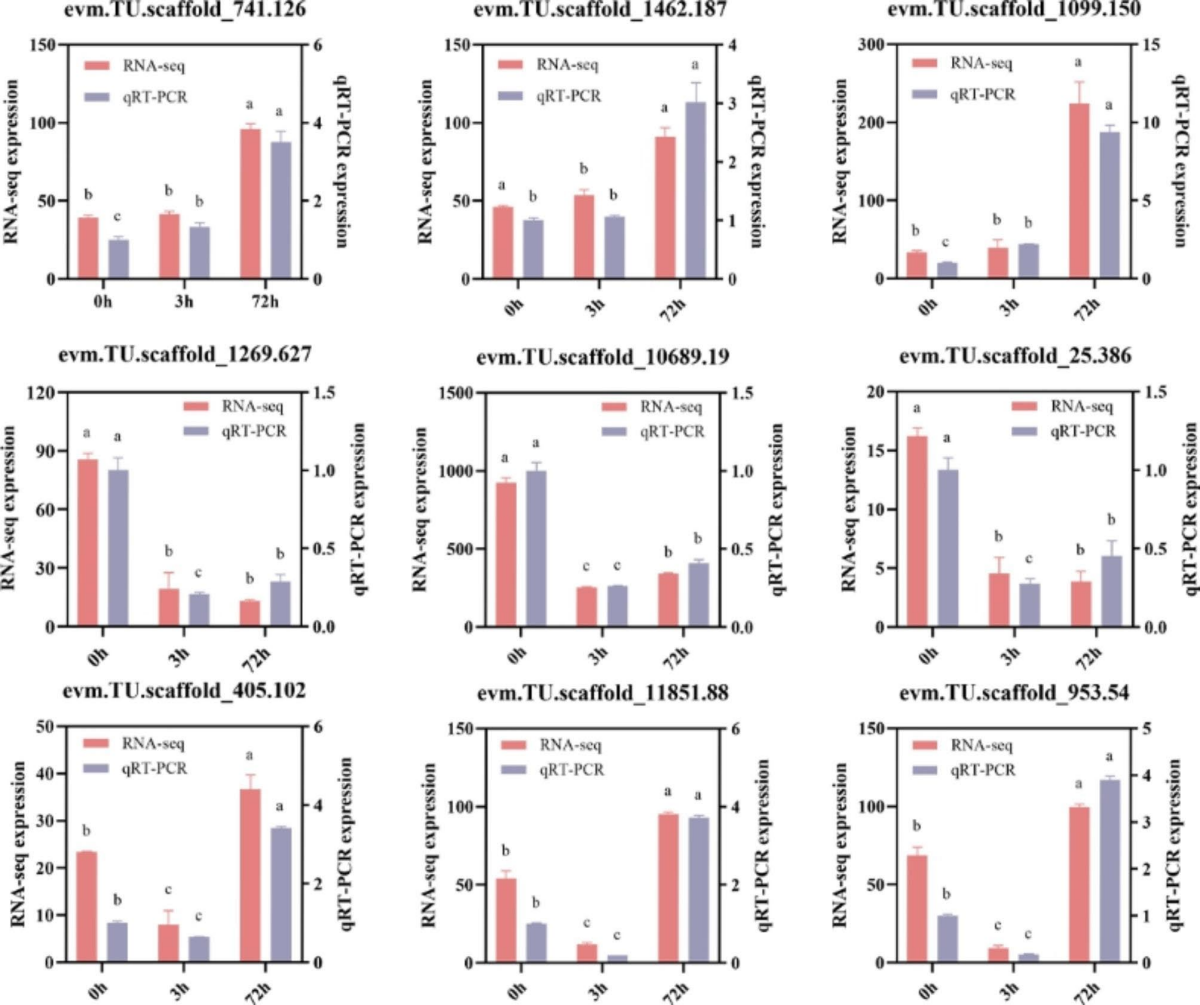
qRT-PCR validation of DEGs. Red for RNA-seq, purple for qRT-PCR. The different letters mean significant differences according to Duncan’s multiple range test at p < 0.05

To confirm the reliability of the RNA-seq data, we verified the expression of DEGs using qRT-PCR. Three DEGs related to secondary metabolism (evm.TU.scaffold_741.126, evm.TU.scaffold_1462.187, evm.TU.scaffold_1099.150), three DEGs related to starch and sucrose metabolism (evm.TU.scaffold_1269.627, evm.TU.scaffold_10689.19, evm.TU.scaffold_25.386), and three DEGs related to proline and arginine metabolism (evm.TU.scaffold_405.102, evm.TU.scaffold_11851.88, evm.TU.scaffold_953.54) were used for validation. The results showed that the qRT-PCR trends were generally consistent with the RNA-seq trends (Fig. 7). These results confirm the reliability and reproducibility of our data.

## Discussion

**Fig. 8:**
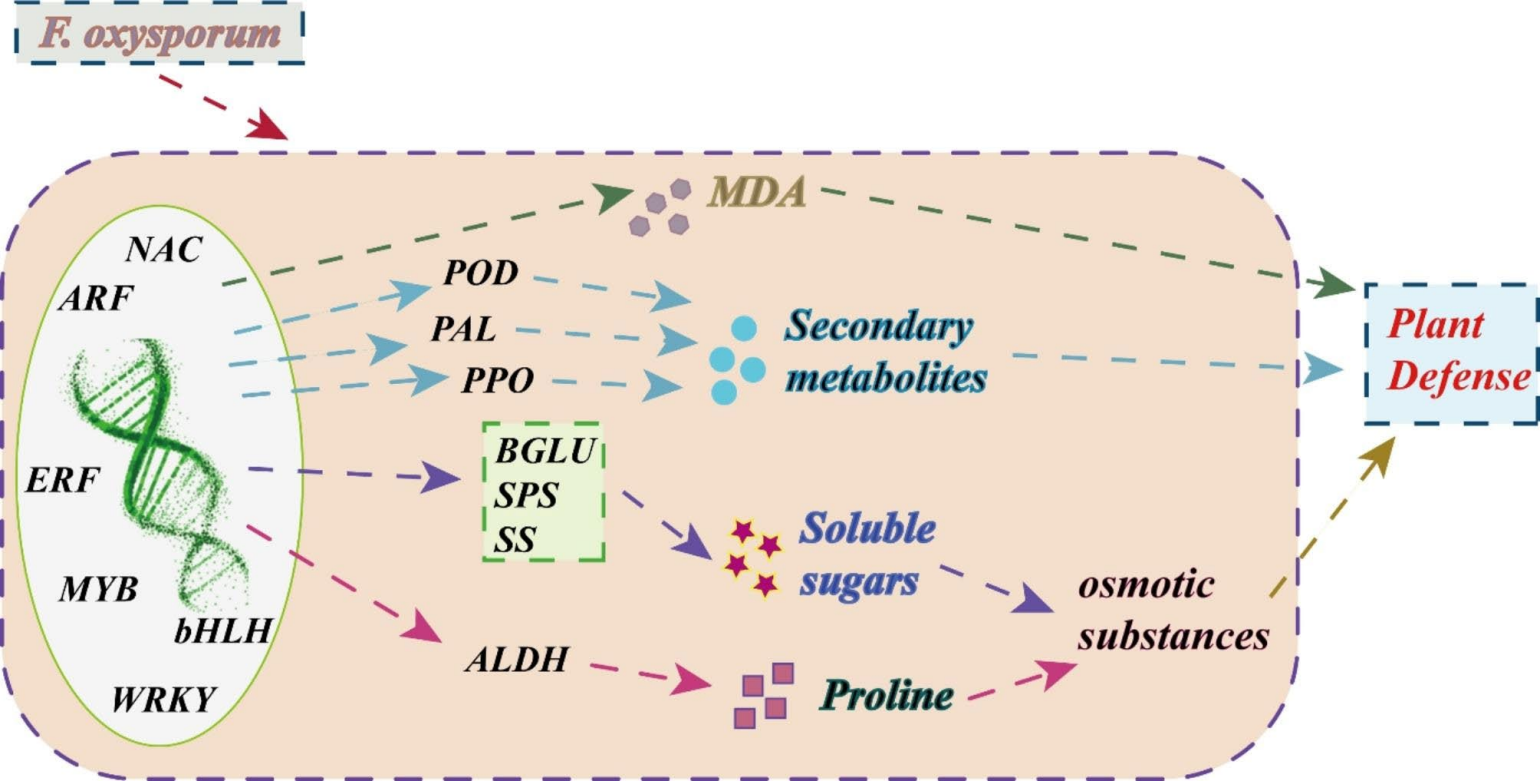
Analysis of the mechanism of ‘Jinba’ response to *F. oxysporum* infection

Due to continuous cultivation, the frequent occurrence of Fusarium wilt disease in chrysanthemums has seriously affected their yield and quality. *F. oxysporum* is the main pathogen causing Fusarium wilt, but there is little research on the molecular mechanism of the chrysanthemum response to *F. oxysporum* infection. In this study, we inoculated ‘Jinba’ with *F. oxysporum* and found that root rot and leaf wilt occurred at 72 h of inoculation (Fig. 1d). In addition, there was a gradual increase in cell membrane permeability and MDA in ‘Jinba’ compared with the control (Fig. 1e), indicating that ‘Jinba’ had started to respond to *F. oxysporum* infection in vivo at the early stage of inoculation. To clarify the molecular mechanism of early response to *F. oxysporum* infection in chrysanthemum, we analyzed root samples from ‘Jinba’ at 0 h, 3 h, and 72 h after inoculation with *F. oxysporum* using RNA-seq. A total of 7985 DEGs were co-expressed between 3 h, and 72 h after inoculation. As our study aimed to mine genes for early response to *F. oxysporum* in chrysanthemum, these 7985 DEGs were candidates for our next analysis using other techniques. We found a significant enrichment of DEGs in response to moisture and biological stimuli, catalytic activity, plant-pathogen interaction, MAPK signaling pathway, starch and sucrose metabolism, and biosynthesis of secondary metabolites using GO and KEGG pathway analysis. We focused our analysis on DEGs in these pathways to provide new insights into the resistance of chrysanthemums to *F. oxysporum* infection (Fig. 8).

Secondary metabolites of plants can act as defenses against plant stress [27, 28]. When infested, plants produce large amounts of phenolic compounds that inhibit the invasion of pathogenic bacteria [29]. For example, α-tomatine limits the growth of pathogens in the apoplast and saponins have strong antifungal activity [30, 31]. Coumaric acid, butyric acid, p-coumaric acid, trans-cinnamic acid, p-hydroxybenzamide, ferulic acid, methyl p-hydroxybenzoate, and 3-indolepropionic acid secreted by *Arabidopsis thaliana* roots inhibit the growth of *F. oxysporum* [32]. Phenylalanine metabolism is one of the most important pathways in plant secondary metabolism. PAL is a key enzyme in the phenylpropanoid pathway that catalyzes the conversion of phenylalanine to cinnamic acid and promotes the production of flavonoids, coumarins, and other secondary metabolites [33]. We found that when *F. oxysporum* inoculated ‘Jinba’, *PAL* gene expression and PAL enzyme activity levels in plants increased. ‘Jinba’ promoted the metabolism of phenylalanine in response to *F. oxysporum*. Caffeic acid O-methyltransferase (COMT) and caffeoyl-CoA-O-methyltransferase (CCoAOMT) are key enzymes regulating lignin biosynthesis [34]. POD and PPO are involved in the synthesis of phenols [35, 36]. Our study showed that *COMT*, *CCoAOMT*, *PPO*, and *POD* gene expressions were upregulated. More importantly, the enzymatic activity levels of PPO and POD were elevated. POD and PPO are also related defense enzymes in plants, which can eliminate oxidative damage by scavenging ROS [37]. The increase in their contents showed that the defense system of chrysanthemum played a role in the early stage of infection, further demonstrating that chrysanthemum responded to the infection of *F. oxysporum* in the early stage of infection. In conclusion, DEGs enriched to the “Biosynthesis of secondary metabolites” pathway involved in the interaction between chrysanthemum and *F. oxysporum*.

Osmotic stress occurs when *F. oxysporum* blocks vascular bundles in plants. When plants are subjected to osmotic stress, they regulate small organic molecules, such as proteins, polyamines, amino acids, betaine, and sugars in their bodies [24]. Soluble sugars can be involved in plant-pathogen interactions in many ways [38]. For example, when *Botrytis cinerea* infects *Arabidopsis*, it inhibits genes related to photosynthesis and promotes sucrose catabolism [39, 40]. When exposed to adverse environments, plants accumulate proline to increase stress tolerance [41]. In addition, Proline is involved in the redox and hypersensitive responses that recognize pathogens [42]. In the present study, we focused on DEGs related to two pathways: “Starch and sucrose metabolism” and “Proline and arginine metabolism”.

β-glucosidase (BGLU) catalyzes the hydrolysis of sugar-containing compounds to release β-D-glucose and the corresponding monosaccharides, oligosaccharides, or complex sugars [43]. We found that during the early stages of *F. oxysporum* infection, the expression of *BGLU* in plants decreased, leading to a reduction in the amount of glucose produced by catabolism in vivo. Sucrose phosphate synthase (SPS) and sucrose synthase (SS) are key rate-limiting enzymes that regulate sucrose synthesis [44, 45]. *SS* family genes and *SPS* were downregulated after inoculation with *F. oxysporum*. Further, the soluble sugar content in ‘Jinba’ gradually decreased after inoculation. We speculate that this may be a mechanism for self-protection in chrysanthemums, as glycogen in the plant can provide nutrients for pathogenic bacteria to infest the plant. When the chrysanthemum experiences invasion by *F. oxysporum*, it inhibits the rate of fungal invasion by reducing sugar synthesis.

In addition, the *ALDH* family encodes pyrroline-5-carboxylic acid synthetase (P5CS), which promotes proline synthesis in proline synthesis [46, 47]. We found that the expression of *ALDH* family genes decreased and then increased after inoculation with *F. oxysporum.* In addition, the proline content in vivo also decreased and then increased. This suggests that proline is involved in the chrysanthemum defense pathway in the early stages of *F. oxysporum* invasion.

In this study, the TFs that responded to *F. oxysporum* infection were mainly in the WRKY, MYB, NAC, ERF, ARF, and bHLH families. TFs, especially the WRKY family, are key members of disease-resistance signaling pathways in plants responding to biotic stress [48]. We found 38 WRKY co-expressed in the present study at 3 and 72 h after *F. oxysporum* inoculation. In addition, we identified a WRKY (evm.TU.scaffold_62.16) with high homology to CmWRKY6 and highly similar conserved structural domains. Song et al. (2014) found that *CmWRKY6* responds to infection by *F. oxysporum* in chrysanthemums [26]. Meanwhile, we analyzed the targeting relationship between evm.TU.scaffold_62.16 and “Plant-pathogen interaction” pathway DEGs. We identified *CPK* as a potential target gene. *CPK* is involved in the plant immune system. In Arabidopsis, *AtCPK1* provides broad-spectrum protection against pathogens by regulating the salicylic acid pathway [49]. In wheat, *WRKY45-1* and *CPK2* may have a common function in multiple disease-resistance pathways [50]. Therefore, evm.TU.scaffold_62.16 or its homologs identified in this study are key candidates for our subsequent analysis of chrysanthemum WRKY in response to *F. oxysporum* infection.

## Conclusions

In conclusion, our study reveals the early response mechanism to *F. oxysporum* infection in chrysanthemum ‘Jinba’. Under the infection of *F. oxysporum*, the levels of MDA, POD, PAL, PPO, and total phenols in chrysanthemum increased in the early stages. Furthermore, with *F. oxysporum* infection, the soluble sugar content in chrysanthemum decreased, and the proline content increased. More importantly, in the early stages, WRKY, MYB, NAC, ERF, ARF, and bHLH were the main TFs responding to *F. oxysporum* infection. We also analyzed the targeting relationship between WRKY and “Plant-pathogen interaction” pathway DEGs to find the candidate WRKY (evm.TU.scaffold_62.16) for our subsequent study. The transcriptomic data and mechanisms revealed in this study can help alleviate the chrysanthemum’s monocropping obstacle, slow the occurrence of Fusarium wilt, and provide new management ideas for the industrial development of chrysanthemum.

## Materials and methods

### Experimental materials

The material for this experiment, chrysanthemum ‘Jinba,’ was provided by Chrysanthemum Germplasm Resource Preserving Center, Nanjing Agricultural University (Nanjing, China) and incubated in a 16 h/8 h (light/dark), a temperature of 25°C, and a humidity of 70% light-temperature chamber.

*F. oxysporum* A2 was isolated from cut flower chrysanthemum ‘Jinba’ plants from the experimental chrysanthemum base at Hushu, Nanjing Agricultural University [18, 19].

### Identification of pathogenic bacteria, culture and inoculation

The isolated *F. oxysporum* A2 was inoculated onto PDA plates and incubated for 5 days at 28 °C. Next, ten cakes of approximately 0.7 cm in diameter were inoculated into 500 mL of PDB and incubated for 4 d at 28 °C, 170 r·min^− 1^ in a constant shaker. The spore morphology and size were observed under a microscope [18, 19]. The genomic DNA of the fungi was extracted using the Biospin Fungus Genomic DNA Extraction Kit, and the extracted DNA was amplified via PCR using primers ITS1F / ITS4 and sequenced [51]. NCBI-BLAST was used for sequence alignment, and Mega X software was used to construct a neighbor-joining phylogenetic tree.

The roots of 40-day-old cuttings of Chrysanthemum seedlings were cut with scissors, immersed in a 10^7^ CFU ml^− 1^ spore suspension for 30 min, and planted in a 1:2 (*v*/*v*) mixture of soil and vermiculite.

### Measurement of physiological indicators

Root samples were collected from plants at 0, 3, and 72 h after inoculation. In addition, enzyme activity in chrysanthemums was measured using POD, PAL, and PPO kits (Comin, Suzhou). In the meantime, the rest of the physiological data were determined using MDA, soluble sugars, proline, and total phenol extraction kits (Jiancheng, Nanjing). The experimental group was inoculated with *F. oxysporum* A2 and the control group was inoculated with PDB.

### RNA extraction and transcriptome sequencing

Root samples were taken from plants at 0, 3, and 72 h after inoculation, and three biological replicates were used for each time point sample. Total RNA was extracted using TRIzol reagent kit (Invitrogen, Carlsbad, CA, USA) and assessed on an Agilent 2100 Bioanalyzer (Agilent Technologies, Palo Alto, CA, USA). After total RNA was extracted, eukaryotic mRNA was enriched by Oligo(dT) beads. mRNA short fragments using NEBNext Ultra RNA Library Prep Kit for Illumina (NEB #7530, New EnglandBiolabs, Ipswich, MA, USA) transcribed into cDNA. Purified double-stranded cDNA fragments were end repaired, A base was added, and ligated to an Illumina sequencing adapter. The resulting cDNA library were sequenced using Illumina Novaseq6000 (Gene Denovo Biotechnology Co., Guangzhou, China). The reads were further filtered using fastp [52]. Finally, paired-end clean reads were mapped to the reference genome using HISAT2 software. 2.4 [53].

The fragment per kilobase of transcript per million mapped reads (FPKM) value was calculated to quantify its expression abundance and variation using RSEM software [54]. The datasets presented in this study can be found in online repositories. The names of the repository/repositories and accession numbers (s) can be found in the NCBI BioProject database under the accession number PRJNA926886.

### DEGs analysis

Correlation analysis was performed using R. Differential expression analysis was performed using DESeq2 [55]. FDR below 0.05 and log2 fold-change ≥ 1 were considered differentially expressed genes/transcripts.

### GO and KEGG Enrichment annotation of DEGs

All DEGs were mapped to the Gene Ontology database (http://www.geneontology.org/) and the Kyoto Encyclopedia of Genes and Genomes (https://www.kegg.jp/) [56–58]. The KEGG related figures for this study have been obtained with permission from the KEGG Pathway database. Hypergeometric tests were applied to identify pathways and GO terms significantly enriched in differential genes compared to the background gene set with FDR ≤ 0.05 as the threshold value.

### Screening of TFs, and analysis of the relationship between *WRKY* and the “Plant-pathogen interaction” pathway DEGs targeting

TFs were annotated using the PlantTFDB (http://planttfdb.gao-lab.org/) database, and statistics were based on TF family classification results. The JASPER database was used to obtain TF binding motif information, and the MEME FIMO software was used to predict transcriptional target genes. Network diagram was drawn to present the association between transcription factors and target genes using Omicsmart (http://www.omicsmart.com).

### Candidate DEG validation using qRT-PCR

The primers for the candidate DEGs were designed using Primer Premier 5, and EF1α was used as the internal reference gene [59]. qRT-PCR was performed using the SYBR Green PCR Master Mix (TaKaRa). qRT-PCR analysis was performed using a Roche LightCycler 96 fluorescence quantification instrument. Each sample contained three biological replicates and three technical replicates were performed for each biological replicate. PCR reactions were performed according to the following reaction conditions: 95 °C for 2 min, 95 ℃ 15 s, 55 ℃ 15 s, 72 ℃ 20 s, 40 cycles. Finally, the dissolution curve program was developed. The relative expression level of each gene was calculated using the formula 2^−ΔΔCT^ [60].

### Statistical analysis

Statistical analyses were performed using SPSS version 25.0. All data were analyzed using analysis of variance (ANOVA) and t-tests to determine significant differences.

## Electronic supplementary material

Below is the link to the electronic supplementary material.

Supplementary Material 1

Supplementary Material 2

Supplementary Material 3

## Data Availability

All transcriptomic sequencing data associated with this study have been submitted to the NCBI SRA under the accession number PRJNA926886 (https://dataview.ncbi.nlm.nih.gov/object/PRJNA926886?reviewer=a5otbqmj0n9lcagjjfje5gnf02).

## References

[1] Hanieh H, Leila S, Abolfazl S (2022). Chrysanthemum, an ornamental genus with considerable medicinal value: a comprehensive review. South Afr J Bot.

[2] Teixeira da silva JA, Shinoyama H, Aida R, Matsushita Y, Raj SK, Chen F (2013). Chrysanthemum Biotechnology: Quo vadis? Critical Reviews in Plant Sciences.

[3] Ma L, Geiser DM, Proctor RH, Rooney AP, O’Donnell K, Trail F, Gardiner DM, Manners JM, Kazan K (2013). Fusarium Pathogenomics. Annu Rev Microbiol.

[4] Beccari G, Hao GX, Liu HQ, Editorial (2022). Fusarium pathogenesis: infection mechanisms and disease progression in host plants. Front Plant Sci.

[5] Li CY, Chen S, Zuo CW, Sun QM, Ye Q, Yi GJ, Huang BZ (2011). The use of GFP-transformed isolates to study infection of banana with Fusarium oxysporum f. sp. cubense race 4. Eur J Plant Pathol.

[6] Bani M, Rispal N, Evidente A, Rubiales D, Cimmino A (2014). Identification of the main toxins isolated from Fusarium oxysporum f. sp. Pisi race 2 and their relation with isolates pathogenicity. J Agric Food Chem.

[7] Aoki T, O’Donnell K, Geiser DM (2014). Systematics of key phytopathogenic fusarium species: current status and future challenges. J Gen Plant Pathol.

[8] Randy CP, Edward AE (2015). The future of global Banana production. Hortic Rev.

[9] Vakalounakis DJ, Chalkias J (2004). Survival of Fusarium oxysporum f. sp. radicis-cucumerinum in soil. Crop Prot.

[10] Tsuda K, Katagiri F (2010). Comparing signaling mechanisms engaged in pattern-triggered and effector-triggered immunity. Curr Opin Plant Biol.

[11] Howden AJM, Huitema E (2012). Effector-triggered posttranslational modifications and their role in suppression of plant immunity. Front Plant Sci.

[12] Peng YJ, Wersch RV, Zhang YL (2017). Convergent and divergent signaling in PAMP-Triggered immunity and effector-triggered immunity. Mol Plant-Microbe Interactions®.

[13] Mandal S, Mitra A (2007). Reinforcement of cell wall in roots of Lycopersicon esculentum through induction of phenolic compounds and lignin by elicitors. Physiol Mol Plant Pathol.

[14] Mandal S, Mitra A (2008). Accumulation of cell wallbound phenolic metabolites and their upliftment in hairy root cultures of tomato (Lycopersicon esculentum Mill). Biotechnol Lett.

[15] Chang CL, Tian L, Ma LN, Li WQ, Nasir F, Li XJ, Tran LSP, Tian CJ (2019). Differential responses of molecular mechanisms and physiochemical characters in wild and cultivated soybeans against invasion by the pathogenic fusarium oxysporum Schltdl. Physiol Plant.

[16] Perochon A, Kahla A, Vranic M, Jia JG, Malla KB, Craze M, Wallington E, Doohan FM (2019). A wheat NAC interacts with an orphan protein and enhances resistance to Fusarium Head Blight disease. Plant Biotechnol J.

[17] Wang LJ, Guo DZ, Zhao GD, Wang JY, Zhang SX, Wang C, Guo XQ (2022). Group IIc WRKY transcription factors regulate cotton resistance to Fusarium oxysporum by promoting GhMKK2-mediated flavonoid biosynthesis. New Phytol.

[18] Miao WH, Ge LJ, Wang YA, Li S, Sun DJ, Liu Y, Guan ZY, Chen SM, Fang WM, Chen FD, Zhao S (2023). Overexpression of CmWRKY8-1–VP64 Fusion Protein reduces resistance in response to *Fusarium oxysporum* by modulating the salicylic Acid Signaling Pathway in Chrysanthemum morifolium. Int J Mol Sci.

[19] Miao WH, Xiao XY, Wang YA, Ge LJ, Yang YR, Liu Y, Liao Y, Guan ZY, Chen SM, Fang WM, Chen FD, Zhao S. CmWRKY6-1–CmWRKY15-like transcriptional cascade negatively regulates the resistance to Fusarium oxysporum infection in Chrysanthemum morifolium, Hortic Res. 2023;uhad101. 10.1093/hr/uhad101 PMC10419886 37577400

[20] Wilhelm BT, Landry JR (2009). RNA-Seq-quantitative measurement of expression through massively parallel RNA-sequencing. Methods.

[21] Su PS, Zhao LF, Li W, Zhao JX, Yan J, Ma X, Li AF, Wang HW, Kong LR (2021). Integrated metabolo-transcriptomics and functional characterization reveals that the wheat auxin receptor TIR1 negatively regulates defense against Fusarium graminearum. J Integr Plant Biol.

[22] Zhang WW, Gao TW, Li PL, Tian C, Song AP, Jiang JF, Guan ZY, Fang WM, Chen FD, Chen SM (2020). Chrysanthemum CmWRKY53 negatively regulates the resistance of chrysanthemum to the aphid Macrosiphoniella sanborni. Hortic Res.

[23] Erb M, Kliebenstein DJ (2020). Plant secondary metabolites as defenses, regulators, and primary metabolites: the blurred functional trichotomy. Plant Physiol.

[24] Holmström KO, Somersalo S, Mandal A, Palva TE, Welin B (2000). Improved tolerance to salinity and low temperature in transgenic tobacco producing glycine betaine. J Exp Bot.

[25] Wani SH, Anand S, Singh B, Bohra A, Joshi R (2021). WRKY transcription factors and plant defense responses: latest discoveries and future prospects. Plant Cell Rep.

[26] Song AP, Li PL, Jiang JF, Chen SM, Li HY, Zeng J, Shao YF, Zhu L, Zhang ZH, Chen FD (2014). Phylogenetic and transcription analysis of chrysanthemum WRKY transcription factors. Int J Mol Sci.

[27] Jan R, Asaf S, Numan M, Lubna, Kim K-M (2021). Plant secondary metabolite biosynthesis and transcriptional regulation in response to biotic and abiotic stress conditions. Agronomy.

[28] Pusztahelyi T, Holb IJ, Pócsi I (2015). Secondary metabolites in fungus-plant interactions. Front Plant Sci.

[29] Boudet AM (2007). Evolution and current status of research in phenolic compounds. Phytochemistry.

[30] Shin M, Umezawa C, Shin T, Batt CA, Tortorello ML (2014). Natural anti-microbial systems | antimicrobial compounds in plants. Encyclopedia of Food Microbiology (Second Edition); publisher.

[31] Trdá L, Janda M, Macková D, Pospíchalová R, Dobrev PI, Burketová L, Matušinsky P (2019). Dual Mode of the Saponin Aescin in Plant Protection: Antifungal Agent and Plant Defense Elicitor. Front Plant Sci.

[32] Walker TS, Bais HP, Halligan KM, Stermitz FR, Vivanco JM (2003). Metabolic profiling of root exudates of Arabidopsis thaliana. J Agric Food Chem.

[33] Huang JL, Gu M, Lai ZB, Fan BF, Shi K, Zhou YH, Yu JQ, Chen ZX (2010). Functional analysis of the Arabidopsis PAL Gene Family in Plant Growth, Development, and response to environmental stress. Plant Physiol.

[34] Vanholme R, De Meester B, Ralph J, Boerjan W (2019). Lignin biosynthesis and its integration into metabolism. Curr Opin Biotech.

[35] Saba MK, Moradi S (2016). Internal browning disorder of eight pear cultivars affected by bioactive constituents and enzyme activity. Food Chem.

[36] And OL, Watson MA (2001). Effects of ascorbic acid on peroxidase and polyphenoloxidase activities in fresh-cut cantaloupe melon. J Food Sci.

[37] Sewelam N, Kazan K, Schenk PM (2016). Global plant stress signaling: reactive oxygen species at the cross-road. Front Plant Sci.

[38] Moghaddam MRB, Ende WVD (2012). Sugars and plant innate immunity. J Exp Bot.

[39] Windram O, Madhou P, McHattie S, Hill C, Hickman R, Cooke E, Jenkins DJ, Penfold CA, Baxter L, Breeze E, Kiddle SJ, Rhodes J, Atwell S, Kliebenstein DJ, Kim YS, Stegle O, Borgwardt K, Zhang C, Tabrett A, Legaie R, Moore J, Finkenstadt B, Wild DL, Mead A, Rand D, Beynon J, Ott S, Buchanan-Wollaston V, Denby KJ (2012). Arabidopsis defense against Botrytis cinerea: chronology and regulation deciphered by high-resolution temporal transcriptomic analysis. Plant Cell.

[40] Ferrari S, Galletti R, Denoux C, De Lorenzo G, Ausubel FM, Dewdney J (2007). Resistance to Botrytis cinerea induced in arabidopsis by elicitors is independent of salicylic acid, ethylene, or jasmonate signaling but requires PHYTOALEXIN DEFICIENT3. Plant Physiol.

[41] Shulaev V, Cortes D, Miller G, Mittler R (2008). Metabolomics for plant stress response. Physiol Plant.

[42] Zeier J (2013). New insights into the regulation of plant immunity by amino acid metabolic pathways. Plant Cell Environ.

[43] Ren RJ, Wang P, Wang LN, Su JP, Sun LJ, Sun Y, Chen DF, Chen XW (2020). Os4BGlu14, a monolignol β-Glucosidase, negatively affects seed longevity by influencing primary metabolism in rice. Plant Mol Biol.

[44] Winter H, Huber SC (2000). Regulation of sucrose metabolism in higher plants: localization and regulation of activity of key enzymes. Crit Rev Plant Sci.

[45] Stein O, Granot D (2019). An overview of sucrose synthases in plants. Front Plant Sci.

[46] Su WH, Zhang C, Wang DJ, Ren YJ, Zhang J, Zang SJ, Zou WH, Su YC, You CH, Xu LP, Que YX (2022). A comprehensive survey of the aldehyde dehydrogenase gene superfamily in Saccharum and the role of ScALDH2B-1 in the stress response. Environ Exp Bot.

[47] Yang ZY, Zhao XC, Shang WN, Liu Y, Ji JF, Liu JP, Tong C (2021). Pyrroline-5-carboxylate synthase senses cellular stress and modulates metabolism by regulating mitochondrial respiration. Cell Death Differ.

[48] Zhang YJ, Wang LJ (2005). The WRKY transcription factor superfamily: its origin in eukaryotes and expansion in plants. BMC Evol Biol.

[49] Coca M, Segundo B (2010). AtCPK1 calcium-dependent protein kinase mediates pathogen resistance in Arabidopsis. Plant J.

[50] Geng SF, Li AL, Tang LC, Yin LJ, Wu L, Lei CL, Guo XP, Zhang X, Jiang GH, Zhai WX, Wei YM, Zheng YL, Lan XJ, Mao L (2013). TaCPK2-A, a calcium-dependent protein kinase gene that is required for wheat powdery mildew resistance enhances bacterial blight resistance in transgenic rice. J Exp Bot.

[51] Song AP, Zhao S, Chen SS, Jiang JF, Chen SM, Li HY, Chen Y, Chen X, Fang WM, Chen FD. The abundance and diversity of Soil Fungi in continuously Monocropped Chrysanthemum. Sci World J. 2013;632920. 10.1155/2013/632920 PMC3821950 24260019

[52] Chen S, Zhou Y, Chen Y, Gu J (2018). Fastp: an ultra-fast all-in-one FASTQ preprocessor. Bioinformatics.

[53] Kim D, Langmead B, Salzberg SL (2015). HISAT: a fast spliced aligner with low memory requirements. Nat Methods.

[54] Li B, Dewey CN (2011). RSEM: accurate transcript quantification from RNA-Seq data with or without a reference genome. BMC Bioinformatics.

[55] Love MI, Huber W, Anders S (2014). Moderated estimation of fold change and dispersion for RNAseq data with DESeq2. Genome Biol.

[56] Kanehisa M, Goto S (2000). KEGG: kyoto encyclopedia of genes and genomes. Nucleic Acids Res.

[57] Kanehisa M (2019). Toward understanding the origin and evolution of cellular organisms. Protein Sci.

[58] Kanehisa M, Furumichi M, Sato Y, Kawashima M, Ishiguro-Watanabe M (2023). KEGG for taxonomy-based analysis of pathways and genomes. Nucleic Acids Res.

[59] Gu CS, Chen SM, Liu ZL, Shan H, Luo HL, Guan ZY, Chen FD (2011). Reference gene selection for quantitative real-time PCR in Chrysanthemum subjected to biotic and abiotic stress. Mol Biotechnol.

[60] Livak KJ, Schmittgen TD (2001). Analysis of relative gene expression data using real-time quantitative PCR and the 2^–∆∆CT^ method. Methods.

