# Supplementary material for: Potential pathways and genes expressed in Chrysanthemum in response to early fusarium oxysporum infection
Source: BMC Plant Biol. 2023 Jun 13;23:312. doi: 10.1186/s12870-023-04331-7 (PMC10262564; doi:10.1186/s12870-023-04331-7)
Supplement: Supplementary file 1 — Supplementary Material 1 [file 12870_2023_4331_MOESM1_ESM.docx]

| Sample | Raw reads | Clean reads | Q20 (%) | Q30 (%) | GC (%) | Mapped reads |
| --- | --- | --- | --- | --- | --- | --- |
| SM-CK-0h-1 | 49,781,718 | 49,482,914 | 96.50% | 90.85% | 42.90% | 38,233,554 (77.61%) |
| SM-CK-0h-2 | 49,678,834 | 49,416,822 | 97.24% | 92.33% | 42.86% | 38,540,344 (78.32%) |
| SM-CK-0h-3 | 49,953,480 | 49,646,880 | 96.69% | 91.23% | 42.76% | 38,388,486 (77.64%) |
| SM-CK-3h-1 | 46,119,926 | 45,826,588 | 97.26% | 92.27% | 42.33% | 38,088,223 (83.17%) |
| SM-CK-3h-2 | 41,720,998 | 41,548,086 | 98.11% | 94.22% | 42.25% | 34,785,440 (83.77%) |
| SM-CK-3h-3 | 41,811,480 | 41,616,918 | 97.73% | 93.28% | 42.50% | 34,192,783 (82.41%) |
| SM-CK-72h-1 | 58,441,672 | 58,156,140 | 97.90% | 93.68% | 42.83% | 46,884,272 (80.73%) |
| SM-CK-72h-2 | 43,151,530 | 42,953,918 | 98.04% | 94.06% | 42.98% | 34,979,979 (81.52%) |
| SM-CK-72h-3 | 46,948,322 | 46,740,020 | 97.93% | 93.78% | 42.93% | 38,066,977 (81.53%) |
| SM-A-3h-1 | 40,956,290 | 40,755,392 | 97.90% | 93.71% | 43.72% | 30,239,427 (74.28%) |
| SM-A-3h-2 | 40,728,722 | 40,522,292 | 98.17% | 94.49% | 45.05% | 27,545,126 (68.12%) |
| SM-A-3h-3 | 35,401,912 | 35,246,216 | 97.98% | 93.92% | 43.61% | 26,186,183 (74.37%) |
| SM-A-72h-1 | 59,810,426 | 59,406,370 | 97.17% | 92.34% | 45.95% | 29,189,945 (49.16%) |
| SM-A-72h-2 | 59,983,222 | 59,587,004 | 97.38% | 92.83% | 46.29% | 27,794,211 (46.67%) |
| SM-A-72h-3 | 59,713,338 | 59,328,212 | 97.14% | 92.24% | 45.87% | 29,102,323 (49.07%) |

**Table S1** RNA-Seq data and corresponding quality control
