# Supplementary material for: Potential pathways and genes expressed in Chrysanthemum in response to early fusarium oxysporum infection
Source: BMC Plant Biol. 2023 Jun 13;23:312. doi: 10.1186/s12870-023-04331-7 (PMC10262564; doi:10.1186/s12870-023-04331-7)
Supplement: Supplementary file 2 — Supplementary Material 2 [file 12870_2023_4331_MOESM2_ESM.docx]

**
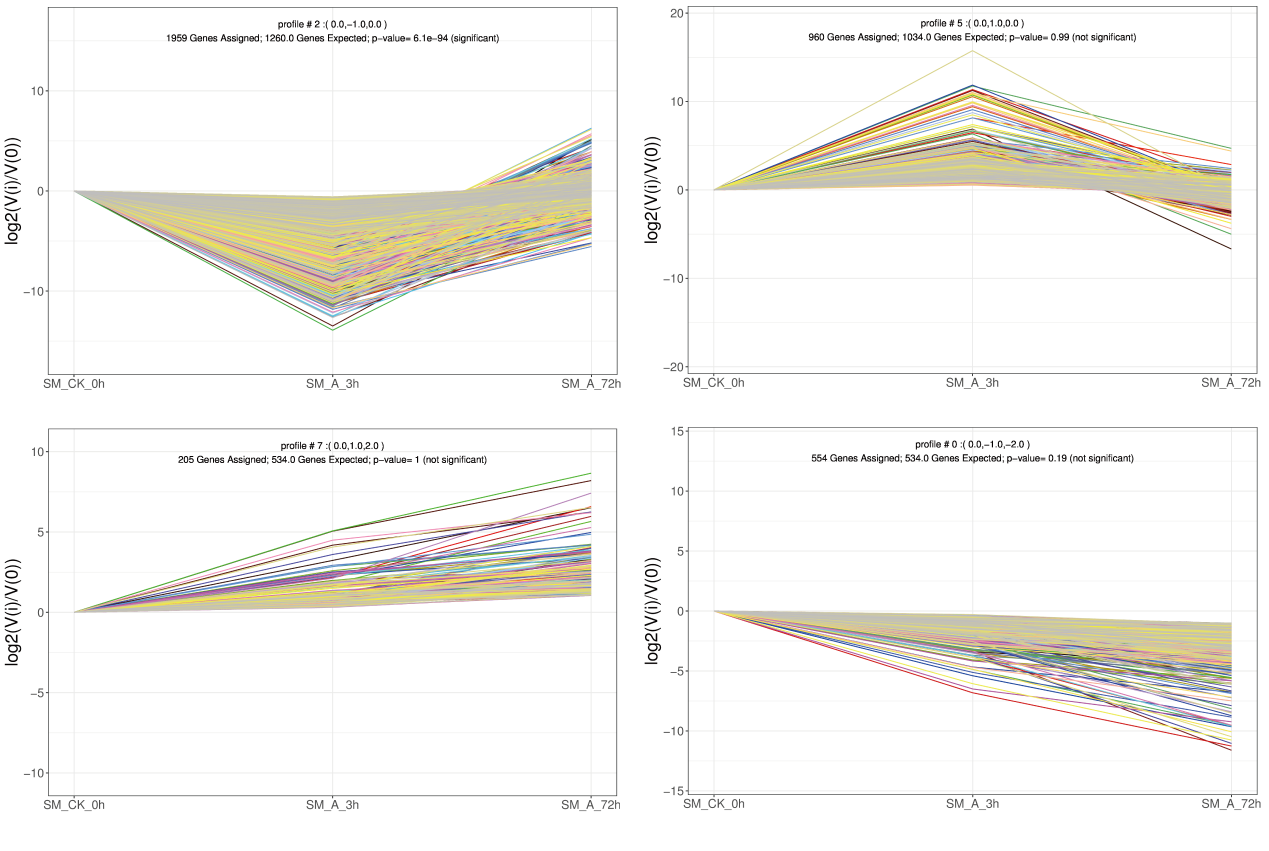
**

**Fig. S1** Expression trend of DEGS.

**
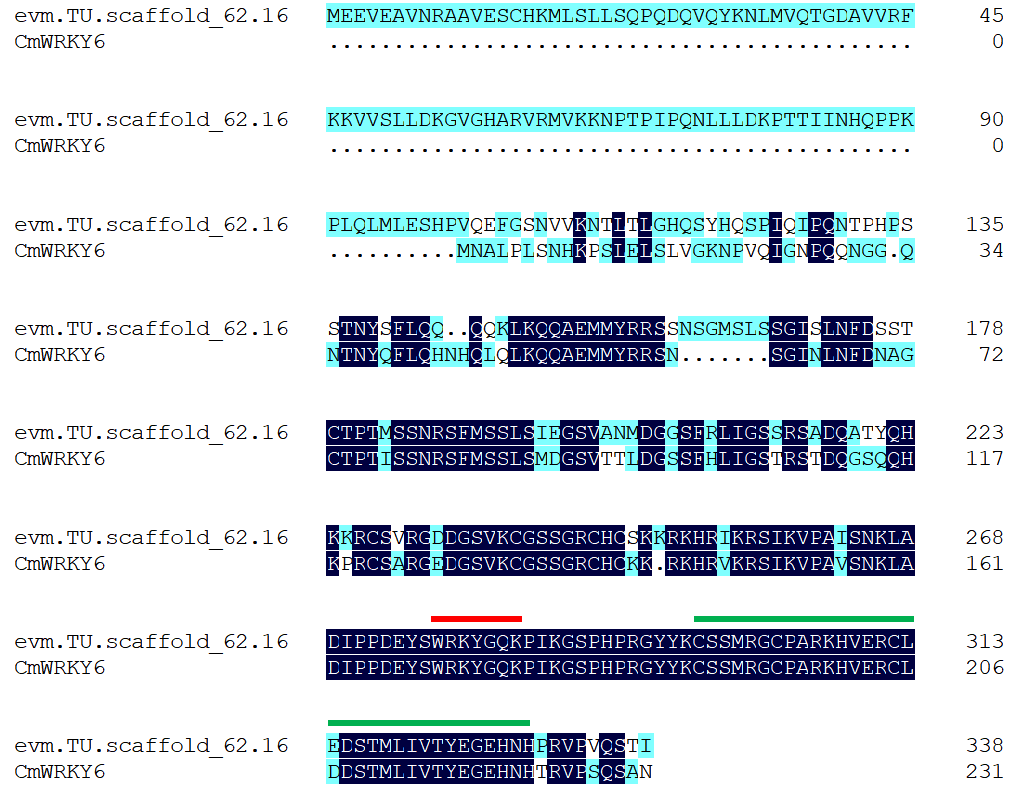
**

**Fig. S2** Sequence comparison of evm.TU.scaffold_62.16 and CmWRKY6. The red line indicates the WRKYGQK structural domain and the green line indicates the zinc finger structural domain.
